# Enterprise modelling for decision-making in the software ecosystem

**DOI:** 10.1038/s41598-025-90916-1

**Published:** 2025-04-01

**Authors:** Anshul Rani, Deepti Mishra, Aida Omerovic

**Affiliations:** 1 Department of Computer Science (IDI), Norwegian University of Science and Technology, Gjøvik, Norway; 2 Software Engineering, Safety and Security, SINTEF Digital, Oslo, Norway

**Keywords:** Computer science, Information technology, Software

## Abstract

Even with the availability of modelling languages such as 4EM and ArchiMate, a noticeable uncertainty persists among professionals regarding the efficient development of process models in the software ecosystem. While current enterprise modelling frameworks and guidelines provide valuable perspectives on essential quality aspects, they are usually more abstract to apply in real-world scenarios directly. This study compares the features of 4EM and ArchiMate, underlining their distinct characteristics for a specific context of software outsourcing. Further, this paper demonstrates a choice of appropriate views of these two modelling languages to model a decision-making scenario for software vendor analysis and selection. Thus, this research supports software enterprises to understand their business processes related explicitly to outsourcing and benefits company personnel involved in decision-making. In addition to model development, 4EM and ArchiMate are tested for the extent they fit for modelling the given decision-making context in the software ecosystem. It is observed that ArchiMate is better suited to the given context. Afterwards, the models are validated using the SEQUAL framework which resulted in identification and incorporation of a new process activity in ‘As-Is’ scenario. This further provided means for digital innovation for ‘To-Be’ scenario of enterprise business process. However, the models developed in this study are premature since they are validated with a theoretical framework only, and the involvement of experts is limited. Thus, more validation studies are required in actual settings with more experts to improve the models further.

## Introduction

The software ecosystem consists of customers, companies, service providers, and stakeholders collaborating on the software of mutual interest^1,2^. Companies outsourcing software requirements perform various activities, which include ‘preparing request for proposal (RFP)’, ‘collecting information of vendors’, ‘processing this vast information’, and finally, deciding on suitable software vendors^2^.

Even though there are various models^3,4^ for decision-making, they often overlook the challenge of defining the decision problem itself, which is vital to analyse the ‘As-Is’ scenario of an organisation in order to identify the need for improvements in the business process and digital innovation^5,6^. Further, there is a need to analyse the sociotechnical conditions within organisational contexts since focusing solely on limited optimisation risks neglects the complexities of organisational reality and its goals^7^.

Given these considerations, it is crucial first to define and formulate the decision problem itself. Although the problem structuring methods^8^ and the group decision-making techniques^9^ emphasise the importance of decision problem definition, these methods lack clear modelling concepts. Modelling language that describes decision processes within an organisational context can serve as a valuable foundation for continuously and systematically analysing and improving decision processes for various contexts and problems^10^. Also, conceptual modelling presents an ‘As-Is’ scenario to identify the areas of improvement in the business processes, allowing an enterprise’s digital transformation^6,11^.

Models, including enterprise (architecture) modelling languages and frameworks, are widely recognised as practical tools to facilitate informed coordination. Various languages and frameworks, including BPMN^12^, UML^13^, ArchiMate^14^, 4EM^15^, have been proposed to create and capture a shared understanding of the desired future state (‘To-Be’ scenario of an enterprise).

Each modelling standard covers different aspects of the problem; thus, a hybrid and integrated modelling approach is recommended^16^. Considering this, ArchiMate is appropriate for high-level business process modelling^14,16^. On the other hand, 4EM follows an abstract yet process-oriented method to represent different aspects of enterprise business processes with an additional advantage regarding the similarity of its notation to traditional modelling languages like UML and BPMN. Although BPMN suits standardisation and automation needs, communicating high-level processes across different teams, organisations, and workflow systems, 4EM’s rich symbols and constructs offer flexibility in capturing interactions between people, objects, roles, and events^15^. Considering the richness and complementary yet similar characteristics of 4EM and ArchiMate are chosen to model the given context of the decision-making process in the software ecosystem.

The objective of this paper is set threefold, considering the gaps described in section "Methodology and related work".

- (1) To define the ‘As-Is’ scenario of the decision-making process in an organisation outsourcing its requirement.
- (2) To assess to what extent ArchiMate and 4EM meet the needs of the given context and how they complement to identify the areas of improvement.
- (3) To reflect on how models can be validated.

The paper is structured into distinct sections for clarity and coherence. Section "Methodology and related work" expounds on the methodology and details of other studies related to the present work, pointing out the research gaps. Section "Context establishment and evaluation criteria" describes the chosen context for modelling and is elaborated upon alongside the established criteria for evaluating and comparing modelling languages. Section "Comparison of modelling languages based on evaluation criteria" provides a comparison between 4EM and ArchiMate is provided based on set criteria. Section "Modelling for decision-making in the software ecosystem" presents the models encompassing an analysis of various perspectives and viewpoints employed in the modelling process. Section "Validation of model" explores using the SEQUAL framework to validate the developed models, while Section "Digital innovation and refined models" briefly outlines the process for assessing digital innovation with refined models. The subsequent section draws concluding remarks and fosters discussions.

## Methodology and related work

This section describes the method and various actions performed to conduct the study, followed by details of related literature to identify the research gaps.

### Methodology

**Fig. 1:**
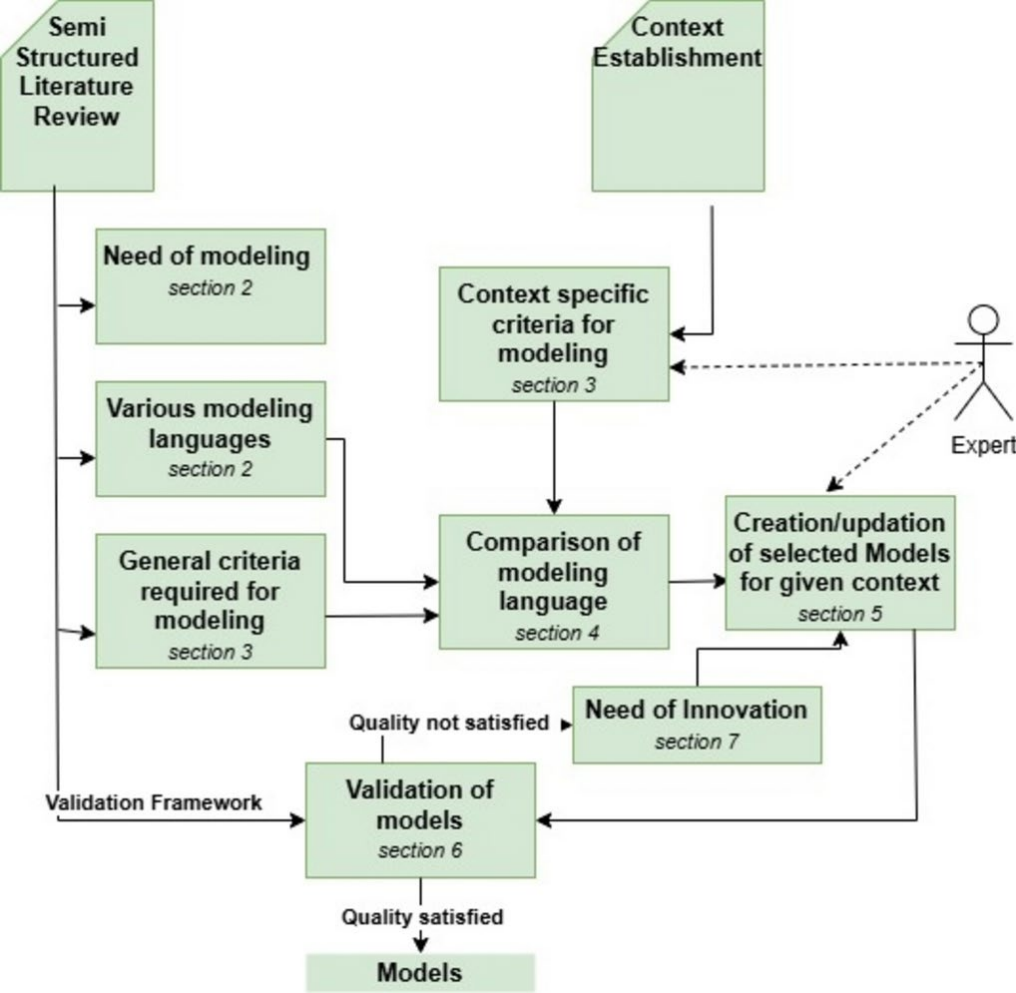
Methodology followed for the study.

Figure 1 shows the method followed for the study. It defines the different stages of the study, from semi-structured literature study, context establishment, model formation, and validation to identifying the need for innovation. A semi-structured literature review is used to get acquainted with various modelling techniques and the need for modelling, along with deriving general parameters for evaluating and comparing 4EM and ArchiMate. Further, languages are compared, and interpretations are drawn based on general and context-specific criteria. Models are created after selecting specific models/perspectives from respective modelling languages based on context-specific criteria in the supervision of experts. Details of experts is given in Table 1. Afterwards, models are validated using the SEQUAL framework, which has been extensively used in literature to validate models^17–20^. Moreover, if the model fails on any of the quality characteristics set by the framework, it is reinvestigated along with the need for innovation.

**Table 1:** Details of the experts.

Expert	Expert Role and Responsibilities	Organisation type	Experience in Decision-making	Location	Expertise
E1	External Consultant for Strategy, Transformation, and Business Architecture	A consultancy Agency	10 years	Norway	Modelling Expert
E2	Decision Maker, Tender preparation and Analysis, Director critical system unit	ICT information and communication technologies	15–20 years	Turkey	Domain Expert

### Related work

Decision support systems (DSS) have been developed by Clark Jr et al.^21–23^ to support decision-makers at various decision-making stages. In this regard, decision analysis methods^4,24^ and model-based DSS^22,23^ are formal ways of creating decision models and optimising different selection variables^10,25,26^. However, these methods still need to provide details on how to model the problem; instead, they only focused on choosing one alternative from many available. Hage^27^ said defining decision problems can be more complex than making the final choice. Thus, ‘*there is a need to define decision-making problem itself and model it’.*

Further, it has been observed that the existing studies^28,29^ primarily focus on specific case modelling, which makes it challenging to adapt to other similar contexts. For example, Kitsios et al.^28^ presented a case of a home business in Greece and showed the relevance of modelling in the given context. However, there is a need to model the software outsourcing problem in general, which can serve as a basis to model decision-making in different domains. Thus, ‘*it is needed that decision-making context itself is modelled for software contexts, i.e., to capture ‘As-Is’ scenario’* which can be adapted for different cases further.

Various modelling frameworks^12–15^ presented in the literature can be utilised for modelling. Tsai et al.^30^ compared different modelling languages and presented the implications of different modelling languages. Unified Modelling Language (UML) is a general-purpose language for software engineering. Some research initiatives use UML for modelling enterprises^31,32^. BPMN is used for business process modelling and focuses on depicting process flows. 4EM is an ontology for describing information system architecture using four entities: data objects, functions, locations, and agents. ArchiMate is a modelling language for enterprise architecture, encompassing business, application, and technology layers. While BPMN and UML target specific aspects of business and software modelling, 4EM focuses on system architecture.

On the other hand, ArchiMate provides a holistic view, aligning business and IT. Anwar and Gill^33^ appraised the suitability and assimilation of high-level and low-level modelling standards for adaptive or agile enterprise architecture modelling. Thus, it is suggested that *‘two modelling languages covering different aspects will represent the modelling and problem thoroughly’*. Also, it is essential to see how context-specific requirements influence the choice of modelling languages and how different modelling languages can complement each other.

Further, it is observed that SEQUAL has been employed to assess and validate various aspects of models and modelling languages. SEQUAL is a tool for evaluating and understanding if the model meets its set objectives^17–20^. It is built on well-established ideas about model quality but has been improved using new theories^20,34,35^ and practical experiences^36,37^ with the original tool. Previously, this included looking at data^38^, ontologies^38,39^, processes^40,41^, enterprises^42^, topological structures^43^, and goal-driven modelling^44^. This framework includes objective (business) aspects of quality, social impact, and subjective quality.

## Context establishment and evaluation criteria

In the software ecosystem, stakeholders of the organisation make decisions and perform various tasks to ensure strategic and architectural goals. Evaluating vendor capabilities and making decisions on vendor selection requires skilled personnel. Managing and evaluating vendors’ capabilities as per the company’s strategic and architectural goals is a subjective task involving processing large amounts of data and creating new artefacts. How is the decision-making process modelled and planned to align with the company’s strategic and architectural goals? What activities do company stakeholders perform to achieve this? Considering these requirements, the following context is set up for modelling along with its scope and beneficiaries as given in Table 2.

**Table 2:** Context establishment for decision-making in the software ecosystem.

Context and Scope	To model activities involved in the decision-making process of organisations/companies with the following scope • Organisations/Companies seeking software requirements to be implemented (through outsourcing) could be a whole new system or part of the existing one • Vendor Management or quality checks of deliverables from selected vendors will not be in the modelling scope
Beneficiaries	• Company personnel involved in the decision-making process of vendor selection, often known as decision-makers • Decision makers could be in various roles, e.g., CEO, Project Manager, Software Architect, Quality Analyst, etc
Entry Criteria	• A company/organisation interested in outsourcing software requirements that constitute a whole or part of the system • The company has decided on the requirements which are to be outsourced
Resources	• Published request for proposals (RFP) • Vendor proposals to be analysed • Decision makers: Different individuals (company personnel or external) involved in the decision-making process Optional: Expert (more details follow in section "Digital innovation and refined models") to support the whole decision-making process by enhancing the capabilities needed to carry out the process
Assumptions	To model the case, a few assumptions are made to keep modelling simple and easy to understand. For example, the models solely reflect on ‘what’ activities are part of decision-making rather than ‘How’ these activities will be carried out

## Evaluation criteria for modelling language comparison

The developed models will be used to understand different activities carried out as a part of an effective vendor selection process. Also, models should be able to reflect on the various aspects that should be incorporated in the vendor selection process so that the process not only becomes easy to follow but also provides practical solutions, i.e., selecting potential vendors. In a nutshell, the purpose is to identify activities followed as a part of the ‘vendor analysis and selection process’ and capture the ‘As-Is’ scenario. Analysing the ‘As-Is’ scenario will lead to innovation to improve or update the processes followed. Also, the model should be clear and complete enough to provide the stakeholders with a step-by-step description of the ‘vendor selection’ process.

The following criteria are considered to compare and evaluate selected modelling languages, i.e. 4EM and ArchiMate. The criteria set for comparison are divided into two categories as follows:

### General criteria

General criteria are extracted from different studies available in the literature, and respective study sources are mentioned along with other criteria.

- **Modelling process**: The modelling process of language should match the enterprise scenario and possibility.
- **Elicitation approach**: The elicitation approach should match the company’s requirements.
- **Abstraction**: It should be able to represent high-level requirements, yet it should be detailed enough so that it is easy to adapt
- **Easy to follow and understand (Understandability)**: Models should clearly explain the vendor selection activities and the different roles responsible for those activities so that users of these models can benefit from them^30^.
- **Ease of adaptation (Adaptation)**: The notations should be easy to follow and self-explanatory for a new user.
- **Able to reflect the need for change/ improvement**: Models should be able to reflect the whole ‘As-Is’ state of the organisation clearly for vendor selection so that the pointers of improvement can be realised toward a more effective process^30^.
- **Community support**: Community support is necessary since it helps new adopters to adapt to the modelling language easily.
- **Tool support**: The available tool support has benefits, and all modelling languages do not have specific tools.

### Context-specific criteria

These criteria are drawn from the context requirements. Extracting these criteria is subjective. The study’s primary purpose is to show how different context-specific criteria can affect the choice of the modelling language and their respective views. Thus, the following context-specific criteria are assumed to carry out the study:

- **Goal/ Vision**: Reflects on the needs/goal of the organisation which participates in outsourcing.
- **Process/ Activity flow**: Presents the activities followed throughout the vendor selection process.
- **Constraints:** Represent barriers and requirements towards the vendor selection process.
- **Actors and resources**^30^: Present the Actors and resources utilised in the vendor selection process so that involved roles can take up the assigned responsibilities and utilise the available resources.
- **Business collaboration**^30^: This shows the external entities the business is collaborating with.
- **Business capabilities**^30^: Notations to represent which business capabilities actors or stakeholders should hold to carry out the decision-making activities.

## Comparison of modelling languages based on evaluation criteria

4EM underscores the significance of harmonising business objectives with IT capabilities to accomplish organisational goals^45^. ArchiMate supports multiple levels of abstraction and furnishes a standardised means of communication and analysis for enterprise architectures. A comparison of both languages based on the set general criteria and context-specific criteria (section "Context establishment and evaluation criteria") is given in Table 3.

**Table 3:** Comparison of 4EM and ArchiMate.

	**4EM**	**ArchiMate**	**Explanation**
Modelling process	Participatory	Participatory/ non-participatory	The modelling process of 4EM is explicitly participatory, involving the different stakeholders. On the other hand, although the modelling process of ArchiMate is similar in its logical progression of steps in which the scope and purpose are first identified, followed by creating, refining, and validating the model, the approach needs to be more explicitly participatory. In other words, ArchiMate may or may not involve all its stakeholders’ participation during the modelling process^15,30,46^
Elicitation approach	Interviews, workshops, surveys, etc	No approach defined explicitly	ArchiMate does not have any explicit set of elicitation approaches defined for it. This is primarily due to the nature of ArchiMate, which placs little emphasis on being participatory ^47,30,46^
Abstraction	Mid-Level	High-Level	4EM is more process-oriented and hence provides more details in comparison to ArchiMate. However, it has more abstraction than a low-level language like UML. Thus, 4EM is considered at the mid-level and ArchiMate at the high level (Bas^46^),
Understandability	Easy	Difficult	ArchiMate is difficult for new adopters, with many notations and views to grasp at once^46^.
Adaption	Easy	Difficult	Special training is needed in the case of ArchiMate; thus, adapting ArchiMate is considered difficult as compared to 4EM (Bas^46^),
Able to reflect the need for change/ improvement	Easy	Easy	With tool support, looking for changes and updates in ArchiMate is easy. (Bas^46^),
Community support	Limited	Extensive	Although 4EM finds use in modelling business processes in enterprises, it is not considered an accepted standard, unlike ArchiMate. The published literature on 4EM is also limited compared to ArchiMate’s, which leads to 4EM having limited community support and fewer practising members^30,46^.
Tool support	No	Yes	The standard tool is not available for 4EM modelling. (Bas^46^),
Goal/Vision	Yes	Yes	Refer to Table 3 for details regarding specific models/perspectives for the representation of these modelling aspects
Process/Activity	Yes	Yes	
Actor view	Yes	Yes	
Constraint	Yes	Yes	
Actor and resources	Yes	Yes	
Business Collaboration	No	Yes	
Business capabilities	No	Yes	

## Modelling for decision-making in the software ecosystem

This section defines the ‘As-Is’ scenario of the decision-making process in an organisation outsourcing its requirements. It details the different views/perspectives chosen from 4EM and ArchiMate to model the various set context-specific requirements (section "Context establishment and evaluation criteria") along with the interpretation that if one modelling language has to be chosen, which one should it be to move further? As stated earlier, the activities and different scenarios are assumed around the established context (section "Context establishment and evaluation criteria") to show the applicability of different aspects of modelling languages.

### Selecting views and modelling ‘As-Is’ scenario

This section presents how 4EM and ArchiMate complement each other along with specific views/perspectives chosen from these modelling languages from all available ones. Table 4 reflects on the different models/ perspectives chosen from these two modelling languages according to set context-specific requirements. It is worth noting that view selection and mapping of context criteria is done with the close collaboration of both experts.

**Table 4:** Mapping context criteria to different views.

Context-specific criteria	4EM	ArchiMate
Goal/Vision	Goal Model	Motivation View
Process/Activity	Process Model	Business Process Model
Actor	Actor and resource Model	*-does not exist-*
Constraint	Business Rules Model	*-does not exist-*
Resources	Actor and resource Model	*-does not exist-*
Business Collaboration	*-does not exist-*	Organisation View
Business Capabilities	*-does not exist-*	Strategy/ Capability View

### Goal/Vision

The goal model focuses on defining and organising specific objectives and tasks to achieve a desired outcome, like creating a project plan for building a website. On the other hand, the motivation view explores the psychological factors driving individuals to work on the website project, such as personal interest in web development or the aspiration to improve their skills, thus understanding what motivates their actions^15^. Combining both approaches can lead to a more comprehensive understanding of the project’s success and the people involved. Thus, for the given context, both views are chosen as stakeholders will be interested in the goals/needs of an enterprise/organisation and the drivers behind them^14,28^.

Assuming that the given context has a primary objective, i.e., to have an effective vendor selection, which is to be realised by sub-objectives: ‘Role out complete RFP’, ‘Having a smooth process to select vendors’ and ‘Reduce conflicts/uncertainties’ during the vendor selection process. Some of these objectives have underlying constraints, e.g., varying perspectives of stakeholders and a lack of standard guidelines.

**Objective 1- Role out RFP**: A complete RFP is a document rolled out to the public that states the requirements to be outsourced. Based on this document, different vendors fill out the proposal and compete during selection. Rolling out a complete RFP is crucial for the vendor selection process, as this is the only document that helps vendors know the requirements of the projects. Accordingly, they showcase their capabilities in the proposals.

**Objective 2—Smooth process to hire vendor:** Companies seek a smooth process to hire a vendor. To the best of our knowledge, there are no standard guidelines for software vendor selection specifically for small and medium organisations.

**Objective 3**—**Reduce conflicts/uncertainties:** Additionally, an organisation seeks that the selection process to be conflict and error-free, as varied opinions and perspectives are considered while making decisions.

**Fig. 2:**
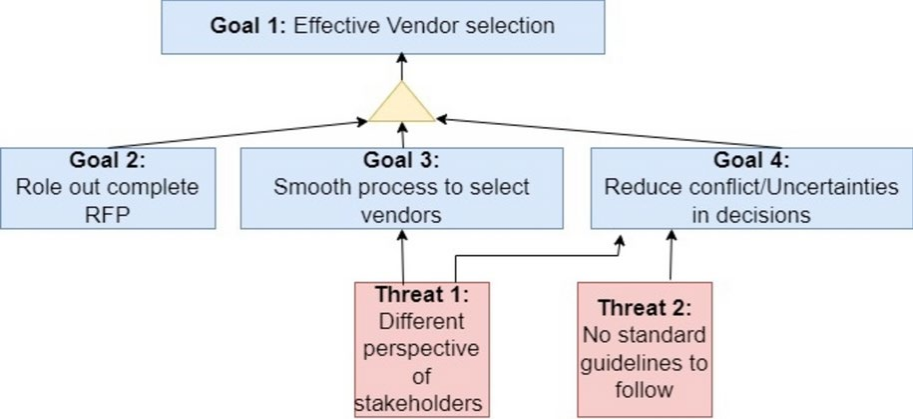
Goal model of 4EM.

**Fig. 3:**
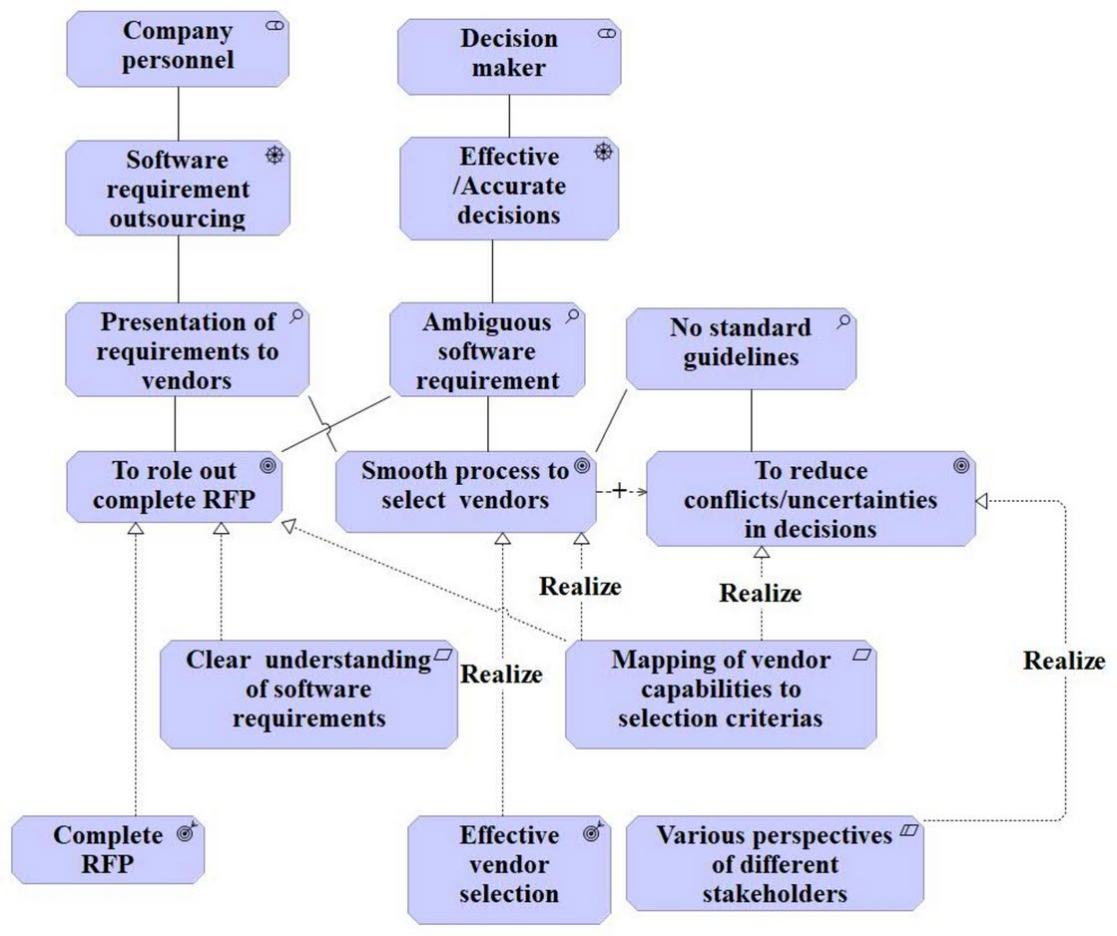
Motivation view of ArchiMate.

Figure 2 and Figure 3 show the goal model and motivation view of 4EM and ArchiMate, respectively. As depicted, the 4EM goal model majorly represents the high-level goals and hinderance in achieving those, if any, named as threats.

The motivation view in Fig. 3 of ArchiMate is similar to the 4EM goal model (Fig. 2). However, the motivation view provides an opportunity to reflect on assertions and motives behind the goals of different roles. As a part of achieving stated goals, i.e., ‘roll out complete RFP’, ‘smooth process to select vendors and ‘reduce conflicts/uncertainties in decisions’, there will be two outcomes, namely ‘complete RFP’ and ‘effective vendor selection’. Mapping of different selection criteria to the vendor capabilities will realise the goal of ‘smooth process of vendor selection’.

Thus, the 4EM view is easy to read and follow to represent high-level objectives, whereas ArchiMate’s motivation view shows the assertions and requirements to reach those goals in detail. The goal model has simple notations, whereas ArchiMate has a variety of notations for different aspects, as explained by Kitsios et al.^28^ and Haren^14^.

### Process/Activity

The Process Model of 4EM focuses on understanding cognition and behaviour about the environment, emphasising how cognition is enacted, embedded, and extended through interaction with the surroundings. It explores the dynamic interplay between individuals and their context, including physical, social, and cultural aspects^15^. On the other hand, the Business Process Model of ArchiMate is designed explicitly for modelling business processes in enterprise architecture. It aims to represent the structure, behaviour, and dependencies of various business processes within an organisation, allowing for better understanding, analysis, and improvement of these processes to achieve organisational goals^14,28^.

**Fig. 4:**
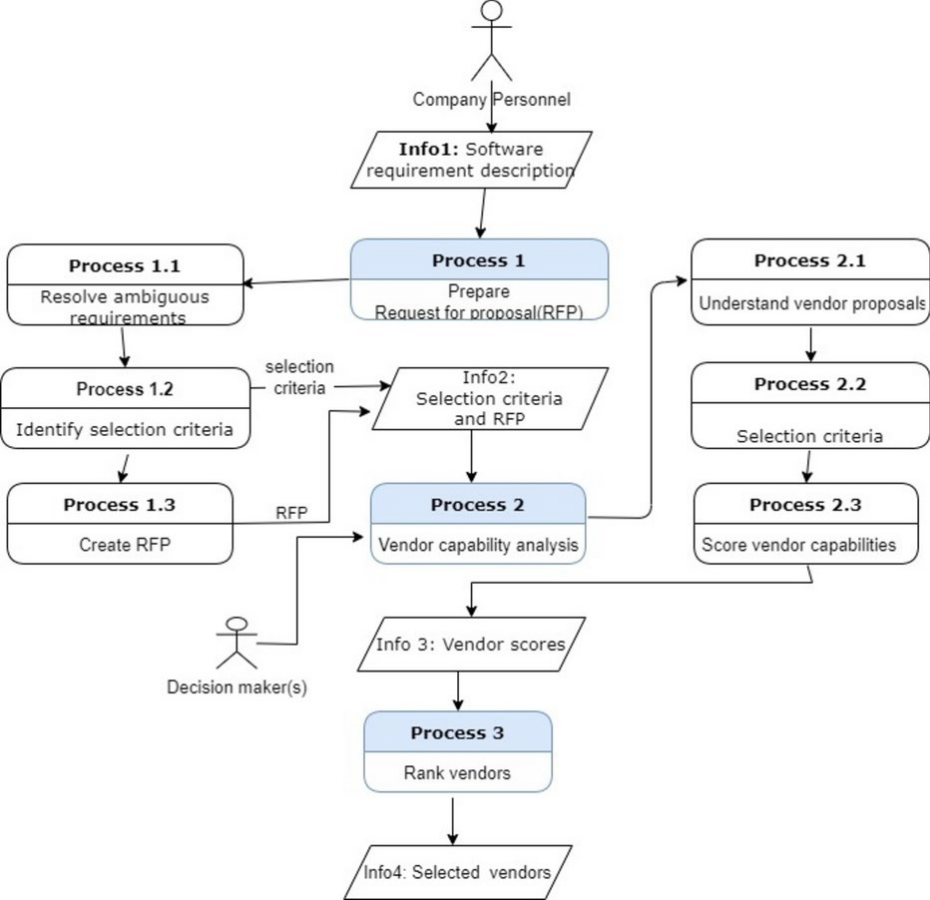
Process model of 4EM.

**Fig. 5:**
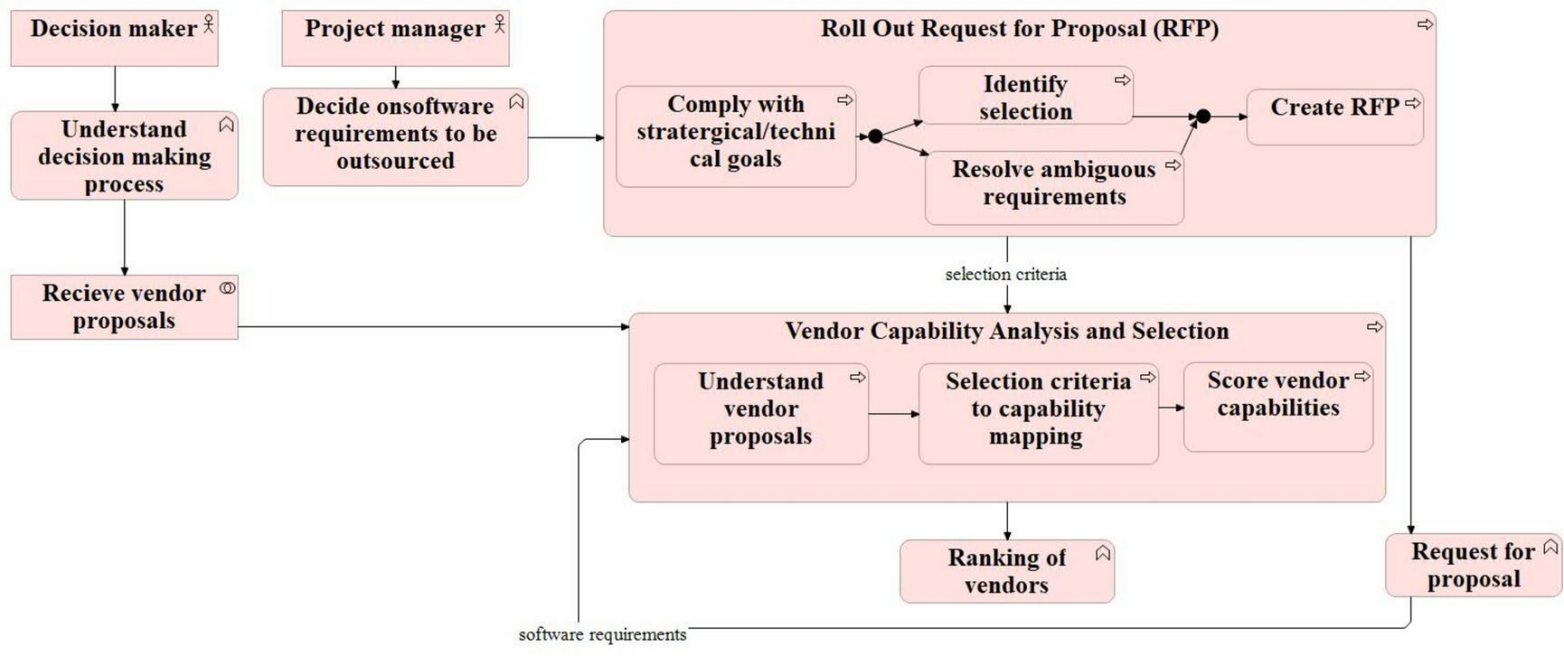
Business Process Model of ArchiMate.

Both models show the processes involved in the ‘effective vendor selection process’. The first process is ‘RFP formation’, which is further realised by several subprocesses from resolving ambiguous requirements and identifying the selection criteria. The ‘RFP formation’ process results in ‘RFP’ and ‘selection criteria’. This information is fed to the second process, ‘vendor capability analysis’. The vendor capability analysis process has further subprocesses, as shown in Fig. 4 and Fig. 5. Lastly, vendors are ranked based on the scores provided as a part of the previous process.

Thus, the Process Model of 4EM provides a broader perspective on cognition and behaviour concerning the environment. In contrast, the business process model of ArchiMate is focused on modelling and analysing business processes from an enterprise architecture perspective. Both 4EM and ArchiMate represent the processes comprehensively. However, again, it is evident that it is easy to understand the notations of the 4EM process model compared to ArchiMate. In contrast, the ArchiMate view provides the facility to represent the subprocess compactly as it is easier to follow and track subprocesses of a process than 4EM.

### Actor and resources

**Fig. 6:**
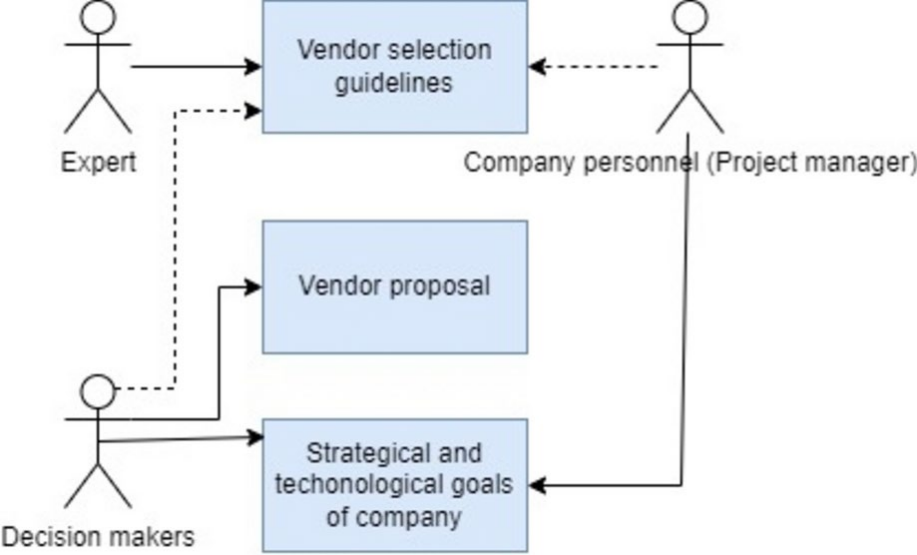
Actor and Resource model of 4EM.

To represent actor and resources utilised for decision-making, the actor resource view of 4EM is chosen. The Actor-Resource View is particularly valuable for understanding the relationships between actors and resources, identifying potential bottlenecks or inefficiencies in processes, and optimising the allocation of resources to improve overall organisational performance. It is often used with other views in enterprise architecture to provide a holistic understanding of an organisation’s structure, processes, and capabilities^15^**,** as given in Fig. 6.

### Constraint

The rule model of 4EM is chosen to represent the constraints. This perspective provides a way to represent mandatory requirements to be fulfilled to reach a particular goal. The rule model represents which rules or mandatory activities are followed by companies as a part of the vendor selection process, which is necessary information to realise the ‘As-Is’ scenario of the companies^15^.

**Fig. 7:**
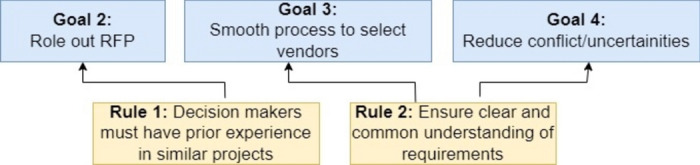
Rule model of 4EM.

Assuming that the primary rule/constraint set by a company is to have all stakeholders/decision makers share a similar level of understanding and knowledge of the complete process of vendor selection (Fig. 7). ArchiMate does not provide an explicit view/ model for the same. However, the motivation view of ArchiMate can incorporate these rules in the form of requirement notations.

Also, the company believes that if people have prior experience, it makes it easier to realise the goals concerning vendor selection. As set by a company to conduct the selection process, these constraints can be represented by the 4EM rule model.

### Business capabilities

**Fig. 8:**
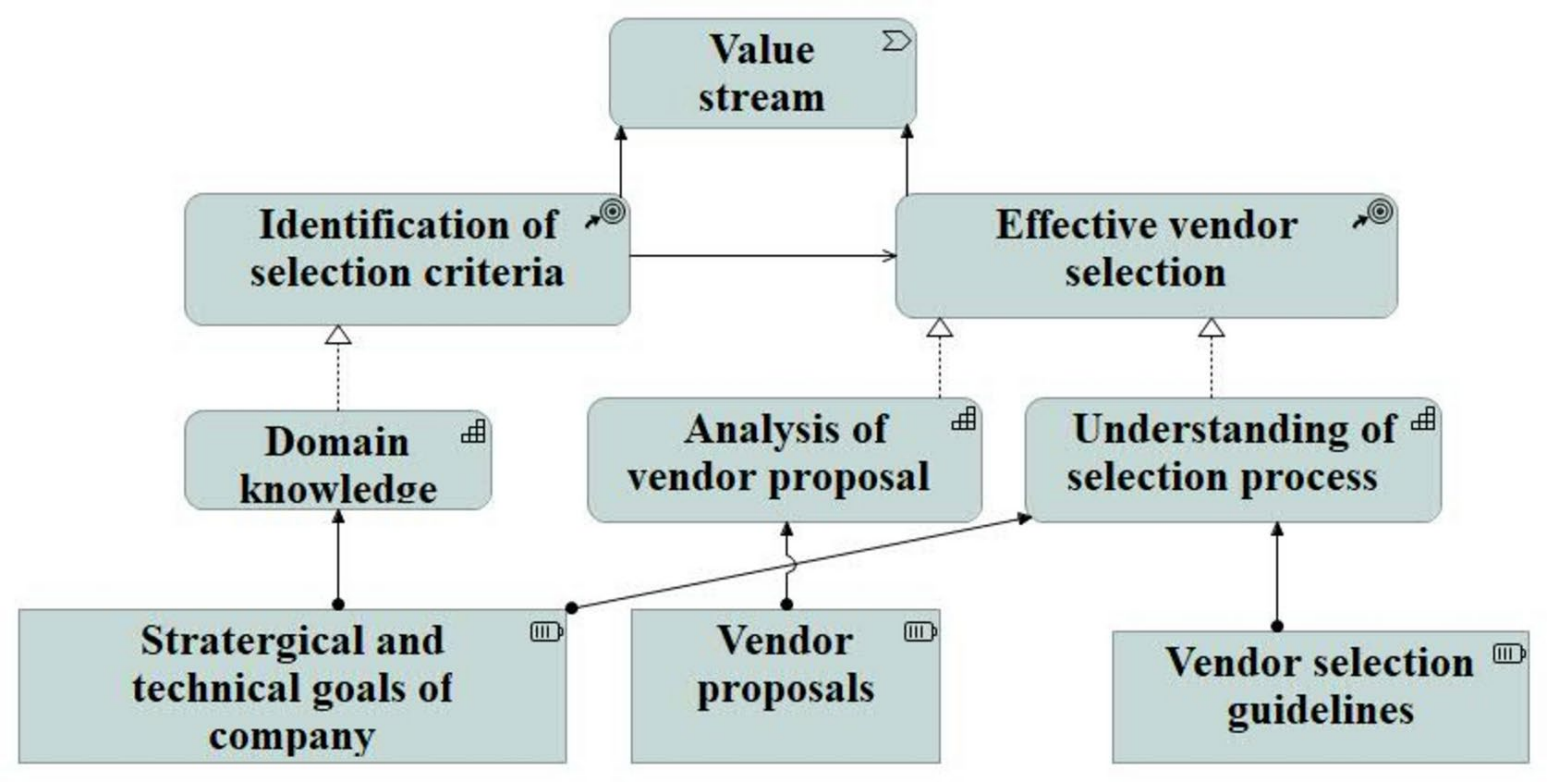
Capability View of ArchiMate.

To show the business capabilities, ArchiMate’s capability view is chosen. This view provides an opportunity to understand the required capabilities and utilised resources to realise the set goals^14,28^. 4EM does not provide any explicit model or view to depict the capabilities and resources required to achieve the outcomes. Whereas the capability view (Fig. 8) of ArchiMate is a subtle representation of outcome connection.

### Business collaboration

**Fig. 9:**
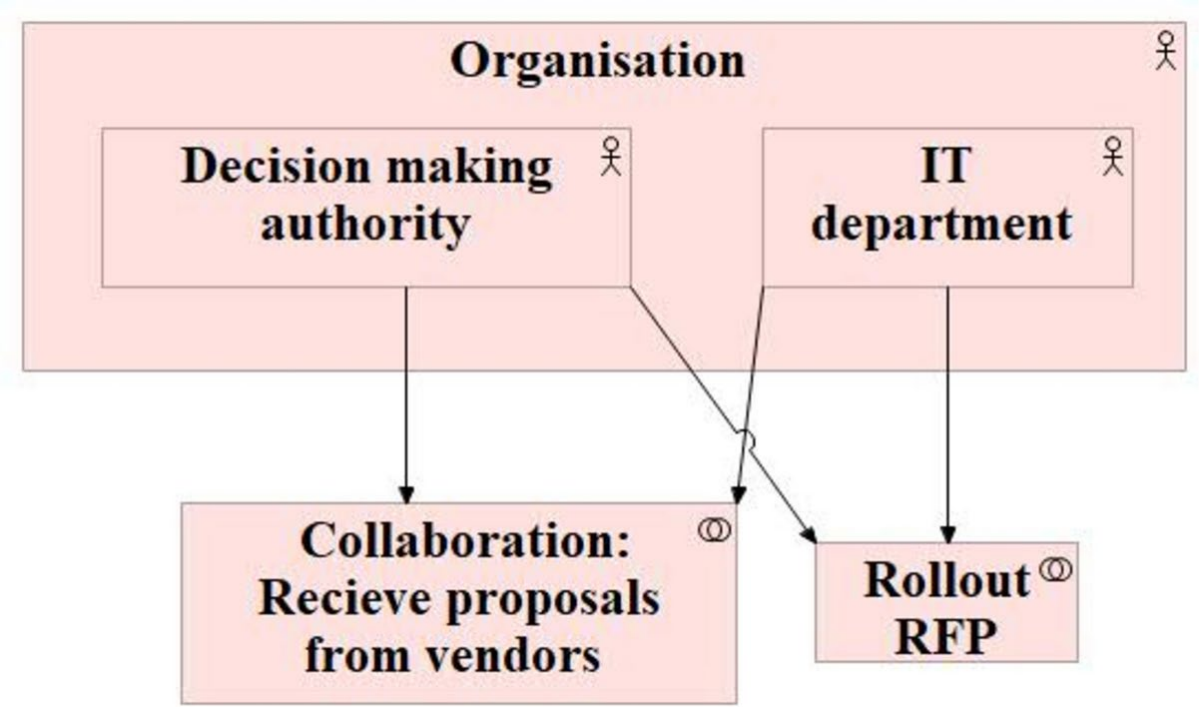
Organisation View of Archimate.

In the given context, the company collaborates with external entities, i.e. vendors. To represent the same, the organisation view (Fig. 9) of ArchiMate is chosen^14,28^.

### Selecting modelling language for validation

Although 4EM and ArchiMate complement each other well, as described in the previous section. Nonetheless, considering the scope of the paper and context requirements, the authors decided to move further with the ArchiMate modelling language for the following reasons.

- 4EM needs more specific representation for decision-making concepts along the way. For example, to represent ‘Goal/vision’, it merely presents ideas for constructing ‘goals’ and ‘subgoals’ (Fig. 2). Within these ‘goals models,’ there is room for incorporating associated concepts like ‘problem,’ ‘cause,’ ‘constraint,’ and ‘motivation’, which is well represented in the chosen ArchiMate motivation view (Fig. 3).
- The business process model of ArchiMate serves the same purpose as 4EM’s process model.
- Information represented by the ‘Actor and Resource model’ of 4EM can be replicated by combining the capability view and motivation view.
- Further, 4EM does not provide an explicit model for modelling the collaboration of an enterprise, which can be realised using the ‘Organisation view’ in ArchiMate.

**Fig. 10:**
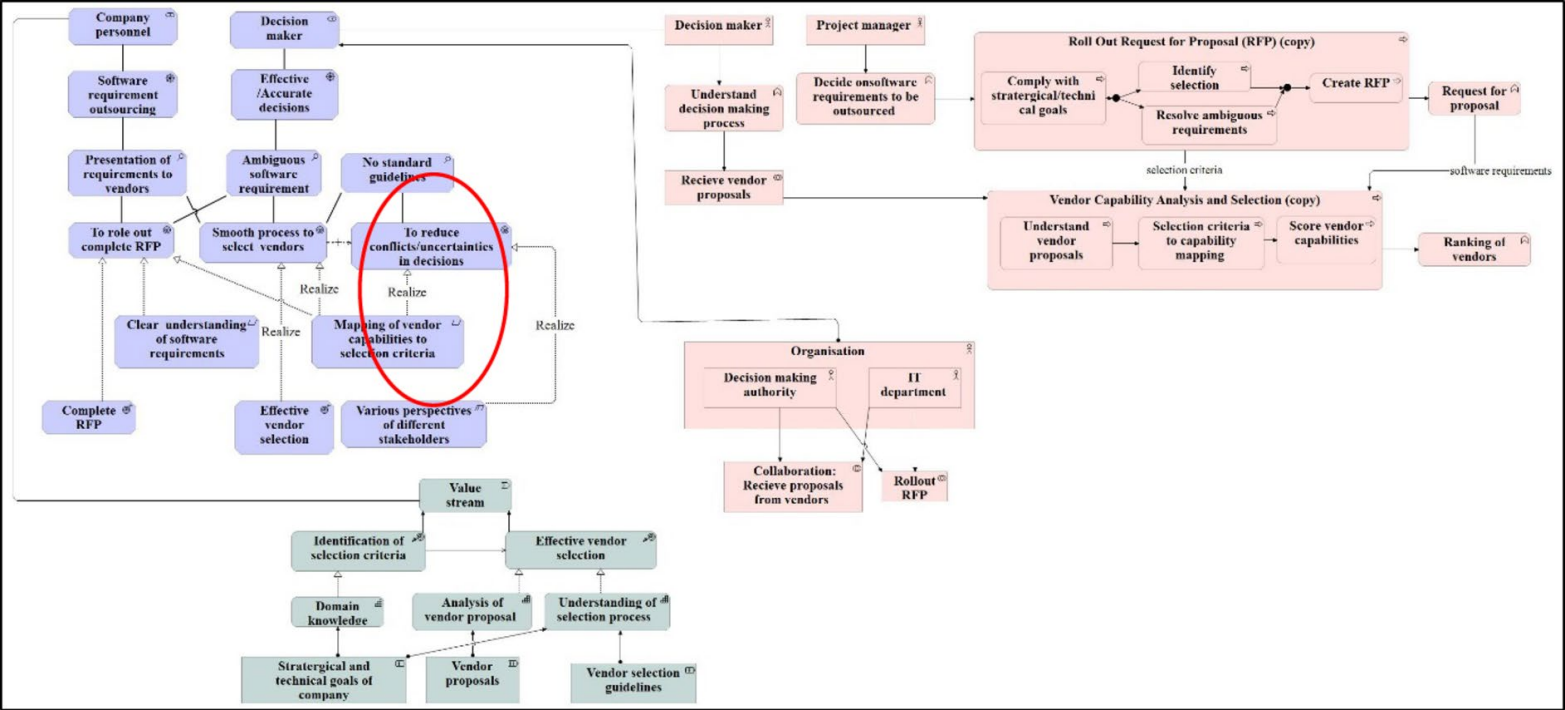
Modelling ‘As-Is’ scenario using ArchiMate for decision-making in the software ecosystem.

Thus, for the given context criteria, ArchiMate is preferred over 4EM. However, the choice of modelling language is subjective and can differ according to scope and context. The complete ArchiMate model for the given context is shown in Fig. 10.

## Validation of model

The modelling aims to understand an enterprise’s ‘As-Is’ scenario and a detailed knowledge of activities involved in efficient vendor selection. This knowledge supports the identification of issues, the enterprise’s goals, and innovation. To assess if the model meets its purpose and to evaluate the models, the SEQUAL framework^48,49^ is used, as suggested in the study by Heggset et al.^50^The framework measures a model’s quality from seven perspectives, as listed below. The experts evaluated the models (Table 1) for different qualities, and hence, the interpretations presented here come from their dialogues. E1, being a modelling expert, could evaluate better models for Physical, Empirical, Synthetic and Pragmatic quality since these qualities are associated with the choice of notation and the modelling language itself. Whereas E2, being domain experts (software outsourcing/ decision-maker) was more comfortable with providing feedback on the Semantic, Social and Deontic quality of model since these quality checks are focused on checking whether models serves the purpose for the set objective or not.

### Physical quality

The physical quality aspect assesses the model’s availability, persistence and whether it is up to date. As the model is usually made for analysis by the company personnel, it should be available for them to innovate. The models are created and stored in the Archi tool (ArchiMate), an accessible web-based program. Also, models can be stored in a local computer or Google Drive. Keeping both copies in synchronisation would take little effort. Thus, the given model (Fig. 10) qualifies the physical quality (according to expert).

### Empirical quality

As per expert model presented in Fig. 10 gives a good overview of current processes, and in general readability is good. However, epert suggested that readability of the enterprise model could be improved by increasing the font size, e.g., by reordering the elements in the model’. This was particularly challenging due to the high number of elements and arrows in the enterprise model in ArchiMate. In addition, it was also appreciated by experts that there are few connections between the different sub-models, which makes the relationship between them easier to identify and thus increases the visibility. However, some of the arrows are quite long, making it harder to read the model. This is considered essential for the readability of the model, even though it has a small negative effect on the empirical quality due to the long crossing arrows. The overall composition of the model (Fig. 10) is good due to colour coding, as suggested by the expert.

### Syntactic quality

The syntactic quality is measured by validity and completeness, which is taken care of using the ‘Archi tool’. ‘Archi tool’ restricts the infeasible connections between different elements. Since the model (Fig. 10) follows the rules of the ArchiMate language, and the syntactic quality is therefore rated good by the experts.

Semantic Quality: The semantic quality measures the model’s validity and completeness. To this E2 appreciated the nomenclature of processes and goals since it makes it evident that they serve the model’s purpose (without knowledge of modelling notations). Additionally, the expert mentioned that all the actors included in the model (Fig. 10) are related to the processes and no components in the model are found redundant since every component represents different sub-requirements which are needed to be fulfilled for decision-making. In other words, the dialogues exchanged with E2 concluded that model and sub models are related to the problem, and thus the model’s validity is good semantically.

Pragmatic Quality: People who read the model (Fig. 10) must also understand it as per pragmatic quality. Using different arrows for different purposes in ArchiMate language and strict notations with no ambiguous meanings make the model entities easy to interpret by the users as mentioned by experts. However, relations between different perspectives can be challenging to understand, but that has been dealt with by providing explanations in running text wherever necessary. Thus, the pragmatic quality can be considered reasonable according to the feedback received.

Social Quality: Social quality assesses whether the stakeholders agree with the model. As users of the models will be business professionals, E2 could interpret the models coming from the related domain, thus, the social quality of model given in Fig. 10 is evaluated as good.

Deontic Quality: The deontic quality measures how well the model coincides with their purpose. The model given in Fig. 10 provides a good overview of the processes and which actors and processes affect the goals. As mentioned by E2, it is hard to follow how the goal ‘reduce error/uncertainties in decisions’ will be realised and which process is responsible (highlighted in red in Fig. 8). To address such concerns, it is essential to trace the patterns and arrows and identify the need for changes^11^.

To conclude, it has been found that no clear evident connections are shown in the model (Fig. 10) regarding which process will realise the goal ‘to reduce conflict/uncertainties in decision’. This goal has the constraint of ‘various perspectives of the different stakeholders’ to be taken care of. This means that the model partly failed the deontic quality. This evaluation leaves us with two questions:

- Can this goal be realised through an existing process? OR
- Do we need an additional process or subprocess to realise it?

Further, this goal has a constraint of ‘various perspective of the stakeholders’, i.e. decision makers for this context. Going through the journey of these users would help in understanding when decision-makers reach a mutual understanding and how various perspectives of decision-makers can be accommodated smoothly.

## Digital innovation and refined models

**Fig. 11:**
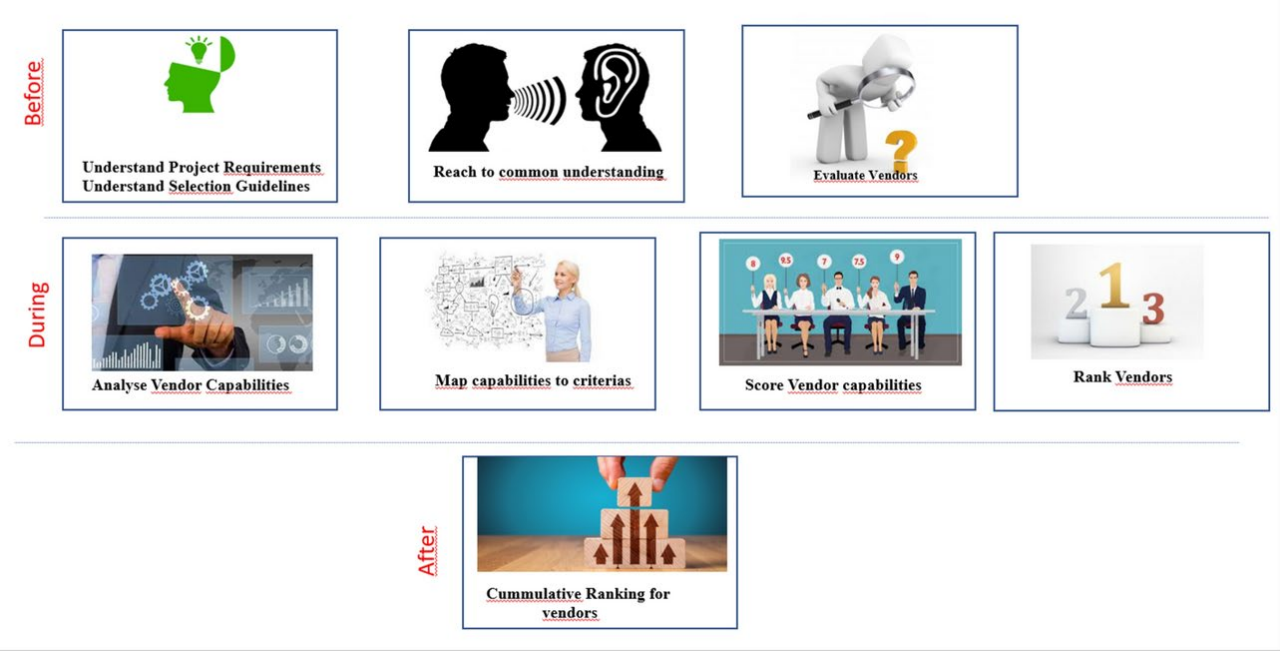
Customer journey of a decision maker before innovation.

Customer journey identifies gaps left in the models and determines how they can be resolved. Figure 11 shows the journey of a decision-maker before any innovation in the models.

**Fig. 12:**
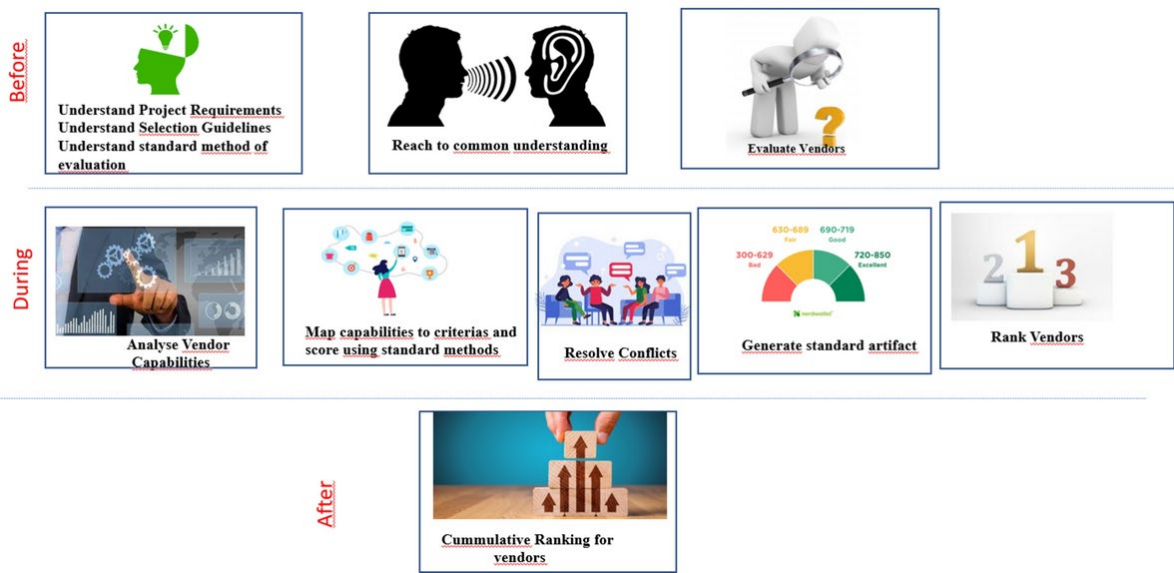
Customer journey of a decision maker after innovation.

From the customer journey, it is found that decision-makers needed a standard method for proposal analysis. Additionally, there are no means to resolve the conflicts if there are any. Thus, after innovation, decision-makers are supposed to follow a common standard way of evaluating and scoring the capabilities (Fig. 12) as part of the decision-making process, leading to process innovation. Afterwards, DMs discuss if any conflicts are found in the scores they provide. The rest of the journey remains the same for the DMs.

**Fig. 13:**
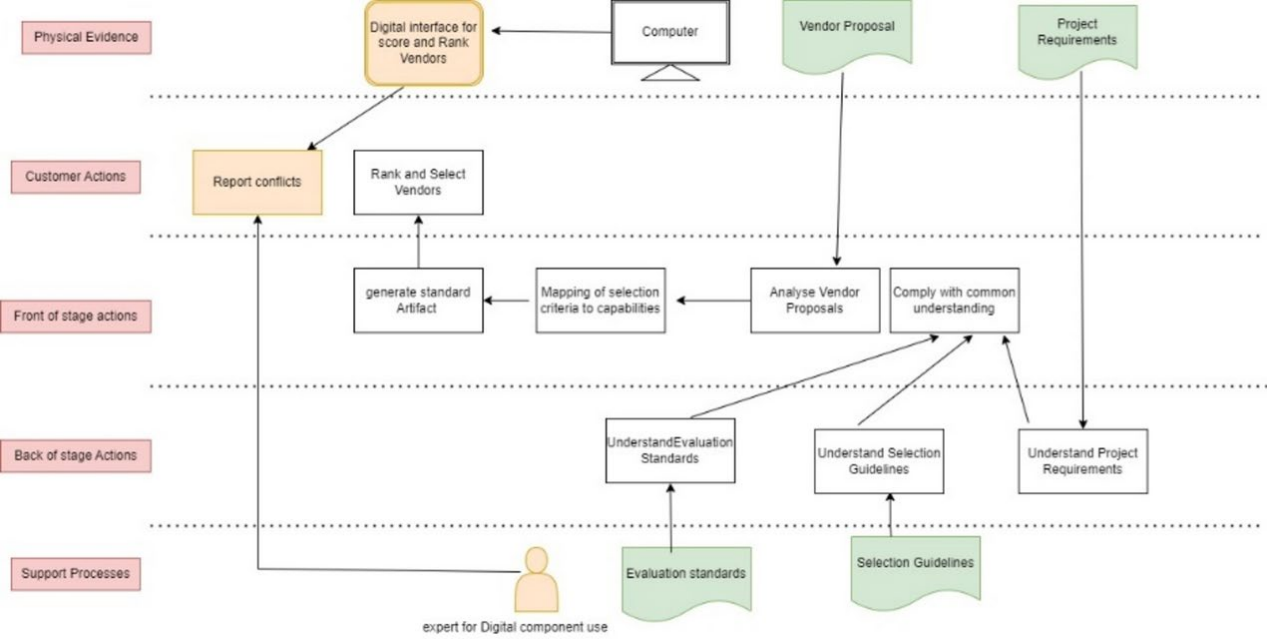
Blueprints designed for digital innovation.

Further, it is observed that identifying the conflicts and generating standard artefacts of ranking manually will be a tedious process until the conflicts are resolved. This gives an opportunity for digital innovation. The blueprint of digital innovation is shown in Fig. 13. Here, we include a digital interface to put in scores of vendors (by different decision-makers) and then rank vendors. This digital interface will also report the conflicts between the scoring of different decision-makers.

Further, making use of digital components requires a special skill set. This skill set can be attained by introducing a new actor named ‘expert’. This can be amongst decision-makers or an external who can guide decision-makers with how to use this digital interface. In the standard format, they will have to score vendor capabilities to identify conflicts and errors.

### Re-designed model

**Fig. 14:**
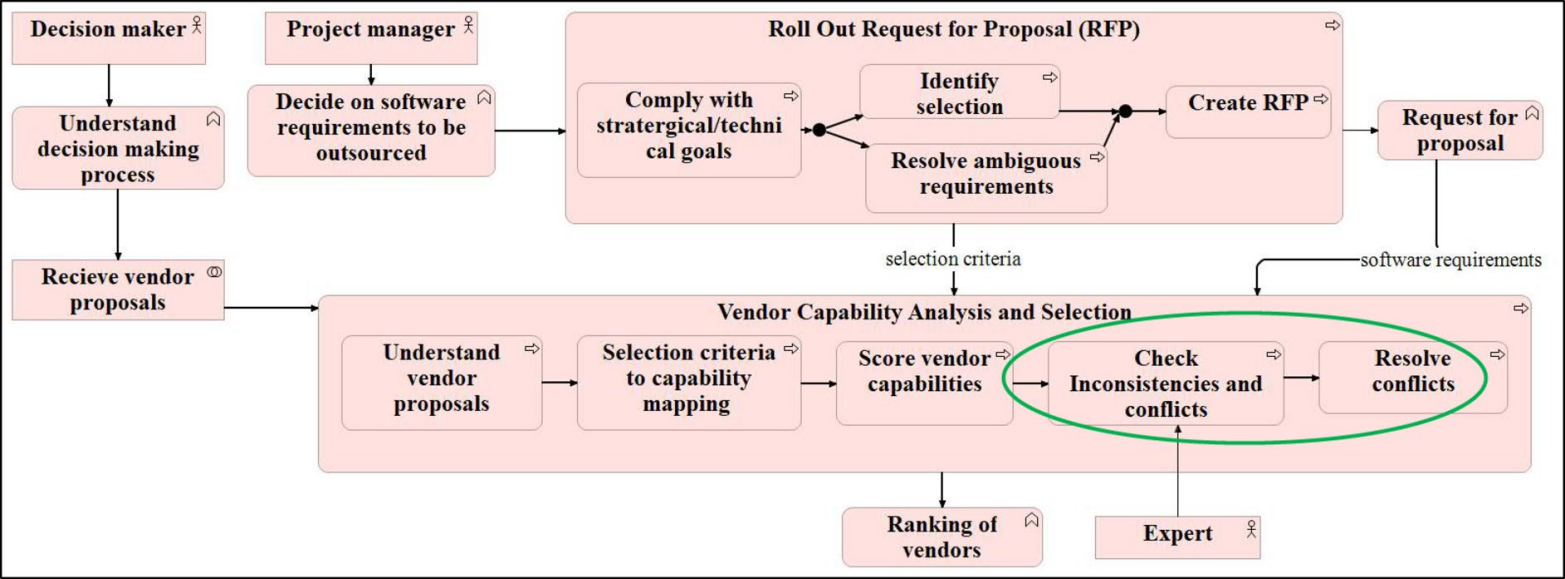
Re-designed business process model after innovation.

As it is process innovation, which suggested a way to realise the goal of ‘reduce conflict and error’, significant change happened in the business process model of the ArchiMate earlier shown in Fig. 5. Subprocess ‘check inconsistencies and conflicts’ and ‘resolve conflicts’ have been added to the process ‘vendor capability analysis’. Also, a new actor ‘expert’ has been included. A new connection has also been introduced between the motivation view and the re-designed business process model (Fig. 14, new connections and additions highlighted with green).

### Validation after innovation

**Fig. 15:**
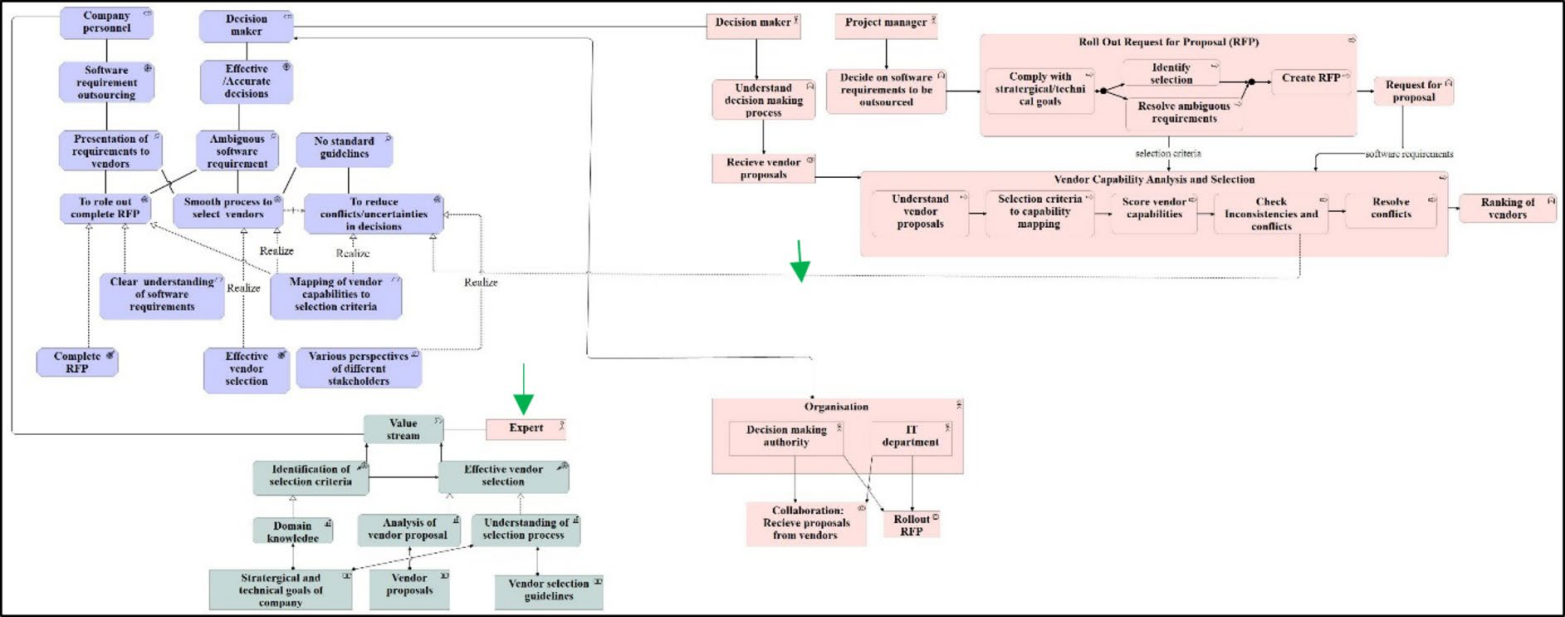
Re-designed enterprise ArchiMate model.

Figure 15 shows the redesigned full ArchiMate model after innovation. This model (Fig. 15) is again evaluated by experts against the SEQUAL framework, as described earlier in section "Validation of model". This model provides a good overview of the processes, and which actors and processes affect the goals. The new updated model passed all the checks, including Deontic Quality.

## Conclusion, implications, limitations, and future work

When businesses encounter productivity, quality, or regulatory compliance challenges, they frequently establish process-aware information systems. However, needing a thorough comprehension of the business processes requiring support can lead to inevitable failure. Creating graphical models to map out business processes is a crucial step in such endeavours. This study supports enterprise businesses by modelling the scenario of decision-making for software vendor analysis and selection in context of software outsourcing. The presented models are generic and can serve as a basis for application-specific problems in similar contexts. However, this study is limited to the software outsourcing contexts only and develops the models for same. Along with model formation, this study compares 4EM and ArchiMate, highlighting their strengths and limitations and showing how different modelling languages complement each other.

Observations from modelling the given context reveal that 4EM notations are notably straightforward to follow, in contrast to ArchiMate, which offers intricate and precise notations for representing various aspects. Furthermore, this study presents a roadmap for selecting different views or perspectives of modelling languages based on the contextual requirements (decision-making in the software ecosystem). The synergy between 4EM and ArchiMate becomes apparent, as 4EM furnishes abstract, high-level information, while ArchiMate offers a more comprehensive approach. Notably, 4EM lacks specific views for depicting business collaboration and capabilities, while ArchiMate efficiently fulfills this role through its ‘capability’ and ‘organization’ views. In conclusion, it’s essential to recognize that the choice of the modelling language and its corresponding views can vary significantly based on the specific contextual requirements one seeks to model.

Decision-making is considered significant for strategic planning to gain a competitive edge in the dynamic and complex interactions of entities in the software ecosystem. This paper offers valuable insights into modelling for researchers who wish to delve deeper into current research topics in business strategy and its modelling. Further, despite modelling languages like 4EM and ArchiMate, professionals need more clarity regarding the effective creation of process models that can be readily comprehended and analysed by analysts and business experts. This paper serves as a practical resource for researchers and practitioners seeking to understand the essential concepts of enterprise modelling and decision-making crucial for successful software outsourcing projects. By modelling the decision-making process in software outsourcing, this study identifies the need for digital innovation for the context under consideration. This study emphasises the scope of digitising some parts of decision-making processes to facilitate effective organisational decision-making. Also, this study highlights the importance of the SEQUAL framework for validating models.

Further, this study has certain limitations that should be acknowledged. Specifically, it represents an initial effort to address the ambiguity and uncertainty inherent in the decision-making process among company stakeholders, particularly in the context of software outsourcing. The decision-making framework presented here is grounded in insights derived from the existing literature, and the models have been developed under expert (limited to two experts) supervision. At this stage, these models have been validated using a theoretical framework; however, to further assess their feasibility and accuracy, future research should incorporate real-world case studies, enabling a more comprehensive evaluation of their practical applicability and effectiveness.

## Data Availability

The models can be made available in their original file format, upon request to the practitioners and research community. Jpg files of models are available at https://zenodo.org/records/14789849. Additional queries can be sent to first author of this paper at anshul.rani@ntnu.no/annuvinayak5@gmail.com.
